# An in-depth investigation of NAs-induced osteoporosis adverse events: a real-world, network toxicology and molecular docking analysis

**DOI:** 10.3389/fmed.2025.1605024

**Published:** 2025-07-04

**Authors:** Jingkai Di, Shuang Wang, Lujia Liu, Likun Qi, Zijian Guo, Yingda Qin, Chuan Xiang

**Affiliations:** ^1^Department of Orthopedics, Second Hospital of Shanxi Medical University, Taiyuan, China; ^2^Shanxi Provincial Key Laboratory of Bone and Soft Tissue Injury Repair, Taiyuan, China; ^3^Department of Gastroenterology, Third Hospital of Shanxi Medical University, Shanxi Bethune Hospital, Shanxi Academy of Medical Sciences, Tongji Shanxi Hospital, Taiyuan, China; ^4^School of Stomatology of Shanxi Medical University, Taiyuan, China; ^5^School of Clinical Medicine, Shanxi Medical University, Taiyuan, Shanxi, China

**Keywords:** adefovir, tenofovir, osteoporosis, G protein-coupled receptor, IL-17

## Abstract

**Background:**

Nucleoside and nucleotide analogs are one of the mainstays of treatment for chronic hepatitis B, but their effects on bone density are highly controversial.

**Methods:**

In this study, four pharmacovigilance analysis methods and Bonferroni-corrected *p*-values were used to analyze the FDA Adverse Event Reporting System database to investigate the relationship between adefovir and tenofovir and osteoporosine-related adverse events. In addition, the biological pathways and target proteins were studied by network toxicology and molecular docking techniques.

**Results:**

Adefovir showed signs of adverse skeletal events at the two PT levels of OSTEOPOROSIS and BONE DENSITY DECREASED, while tenofovir showed signs of adverse skeletal events at the five PT levels of BONE DENSITY DECREASED, BONE LOSS, OSTEOPENIA, OSTEOPOROSIS and OSTEOPOROTIC FRACTURE. Furthermore, at the overall SMQ level, positive signals of adverse skeletal events were also valid. Subgroup analysis showed that adefovir was more likely to cause osteoporosis in the elderly and women, while tenofovir exhibited the opposite trend. Furthermore, GO and KEGG analyses indicated that both drugs may jointly promote osteoporosis through pathways such as cell migration, G protein-coupled receptor and Toll-like receptor signaling pathways. Molecular docking technology further reveals that the two drugs can produce pathological effects by binding to osteoporosis-related genes such as ADORA1 and JAK1.

**Conclusion:**

This study comprehensively reported the risk and mechanisms of osteoporosis caused by the clinical use of NAs drugs, and provided more detailed recommendations for clinical improvement and prevention of adverse events.

## 1 Introduction

Chronic hepatitis B (CHB), a chronic inflammatory disease of the liver caused by hepatitis B virus (HBV) infection, has a large patient population (1). According to the World Health Organization, by 2022, 254 million people worldwide will have CHB, with up to 1.2 million new infections each year, making it one of the world’s major health problems (2, 3) As the disease continues to progress, patients with CHB will face the development of advanced liver disease such as liver cirrhosis and liver cancer (4). In addition, the total medical cost of hepatitis B related diseases accounts for 151.6% of the annual income of patients’ families, and the annual treatment cost exceeds 700 million US dollars, which seriously affects the quality of life of patients and brings a huge burden to families and society (5, 6).

The primary treatment for chronic hepatitis B (CHB) is nucleoside and nucleotide analog (NAs) therapy (7). They are the first-line choice for the treatment of CHB, mainly by inhibiting the activity of HBV deoxyribonucleic acid polymerase, which in turn exerts a good antiviral effect (8, 9). Currently, the NAs commonly used in clinical practice are entecavir, adefovir, telbivudine and tenofovir (10, 11). Among them, adefovir and tenofovir are representative of the first- and second-generation anti-hepatitis drugs, respectively, so this study will focus on adefovir and tenofovir (12).

It is worth noting that there is still controversy about the adverse effects of Adefovir and Tenofovir, and their effects on bone health are high on the list of points of contention (13). Long-term use of both Entecavir and Tenofovir disoproxil fumarate is associated with an increased risk of bone and kidney damage, according to an analysis of 211 patients with CHB who received entecavir monotherapy (13). Another retrospective study similarly noted that long-term use of low-dose Adefovir for the treatment of hepatitis B may result in bone pain accompanied by adverse effects such as hypophosphatemia and elevated alkaline phosphatase (14). However, the adverse effects of Adefovir and Tenofovir on bone health are not widely recognized. In some cases, patients with chronic hepatitis with osteoporosis have instead experienced improved bone safety with Tenofovir (15). In addition, a retrospective study with a four to five years follow-up found that patients with CHB treated with tenofovir disoproxil fumarate or entecavir did not have a significant increase in the incidence of bone loss and osteoporosis (16). Therefore, there is an urgent need for real-world evidence to clarify the specific link that exists between Adefovir and Adefovir and bone health.

The FDA Adverse Event Reporting System (FAERS) database is the world’s largest self-reported adverse event database designed to help the FDA better monitor the post-market safety of drugs and therapeutic products (17, 18). In addition, emerging network toxicology translates complex multi-component, multi-target toxicity pathways into intuitive graphical representations that systematically reveal how target toxins trigger pathological mechanisms (19). Molecular docking analysis predicts ligand-protein binding capacity and binding sites, and identifies relevant core active ingredients, providing molecular docking for key targets and actions to further explore the potential mechanism of action of drugs (20).

Therefore, using the FAERS database and molecular docking analysis, the present study focused on the real-world bone health adverse effects profile of Adefovir and Tenofovir esters and clarified the specific mechanisms involved, with the aim of providing a theoretical basis for the rational use of anti-hepatitis virus medications in the clinic and for the prevention and management of associated bone health risks.

## 2 Materials and methods

### 2.1 Real-world data analysis

#### 2.1.1 Data sources

FAERS is a publicly available database containing data on adverse events (AEs) and medication errors that are spontaneously reported to the FDA. Of these, Individual Case Safety Reports (ICSRs) from Q1 2004 to Q3 2024 were included in this study and were subjected to pharmacovigilance studies. The FAERS dataset used consists of seven data tables and includes demographic information (DEMO), Drug Information (DRUG), Adverse Event Codes (REAC), Patient Outcomes (OUTC), Reporting Sources (RPSR), Treatment Start and End Dates Associated with Reported Drugs (THER), and Indications for Medication Administration (INDI). The database identifies the content of each record by PRIMARY ID. To minimize data bias, we performed case deduplication prior to statistical analysis according to the FDA-recommended methodology for removing duplicate reports. In addition, subsequent case reports in the FAERS database may contain updated information on the initial case report, so we needed to remove redundancy in this part of the dataset based on a combination of the following six fields: event date, age, gender, adverse event, drug group administered, and country of report, and to select the most recent record from the available cases. As FAERS is a public database containing de-identified data, ethical approval was not required.

#### 2.1.2 Standardized definition of adverse events

Suspected adverse reaction data in the FAERS database were categorized using the Preferred Terminology (PT) levels in the Medical Dictionary for Regulatory Activities (MedDRA) version 26.1. The Standardized MedDRA Queries (SMQs) is a comprehensive, proven, predefined set of preferred terminology used to assist regulators and pharmaceutical companies with drug safety issues (21). PT is a term reserved for the specific expression of a single medical concept such as a symptom, sign, disease, diagnosis, indication, examination, surgical and medical operation, medical, social or family history (22). The 10 PTs associated with osteoporosis (“bone density decreased,” “bone formation decreased,” “bone loss,” “bone marrow oedema syndrome,” “osteopenia,” “osteoporosis,” “osteoporosis postmenopausal,” “osteoporotic fracture,” “resorption bone increased” and “senile osteoporosis”) were included in the study and identified in the database. In addition, drugs in the FAERS database are reported using four classifications that designate the role of the drug for the reported adverse event: primary suspect drug, secondary suspect drug, concomitant drug, and interacting drug. We only considered reports labeled as primary suspect drugs based on the “role_code” field.

#### 2.1.3 Statistical analysis

Pharmacovigilance studies were conducted by disproportionate analysis to identify potential drug-adverse event associations after removing duplicate data. Based on disproportionality analysis, four methods of Reporting Odds Ratio (ROR), Proportional Reporting Ratio (PRR), Information Component (IC) and Empirical Bayesian Geometric Mean (EBGM) were used for the study. These four methods were used to detect signals, and according to the pharmacovigilance consensus, the adverse event was considered drug-related when at least one of the signals in the algorithm met the requirements (18). See Supplementary Table 1, for specific equations. The Weber distribution test describes the risk that the AE will increase or decrease over time. Its shape parameter *β* determines the shape of the distribution function. When the shape parameter *β* is less than 1 and its 95% CI is less than 1, the risk of adverse events is considered to decrease over time (early failure curve). When the shape parameter *β* is equal to or close to 1 and its 95% CI includes a value of 1, adverse events continue to occur over time (random failure curves). Finally, when the shape parameter *β* > 1 and its 95% CI value does not include 1, the incidence of adverse events is thought to increase over time (wear failure curve) (23). The data were organized and statistically analyzed in this study using R (version 4.4.1) and the corresponding version of Rstudio.

### 2.2 Network toxicology

#### 2.2.1 Screening of drug targets

3D structure and SMILES codes were obtained from PubChem database^1^ (24). Gene targets of adefovir and tenofovir were screened from Binding DB^2^ (25), Comparative Toxicogenomics database (CTD)^3^ (26), and TargetNet^4^ (27).

#### 2.2.2 Weighted gene coexpression network analysis (WGCNA)

GSE56814 analyzed for this study was downloaded from NCBI Gene Expression Omnibus (GEO) database. This dataset contained gene expression data of blood mononuclear cells from 80 participants, including 40 women with high bone density and 40 women with low bone density. WGCNA analysis was performed to screen the modules which were significantly associated with osteoporosis. Cluster analysis was performed to detect samples with outliers, which should be removed from the subsequent analysis. The “soft” threshold power (*β*) and scale-free network coefficients were computed to construct a scale-free network. The modules significantly associated with osteoporosis were then identified. The minimum of genes per module was set to 30. The intersection of significant module genes and drug targets was employed to identify potential gene targets for drugs influencing osteoporosis.

#### 2.2.3 Enrichment analyses

To explore the significantly enriched biological pathways involved in the intersection of adefovir, tenofovir and osteoporosis-related genes, the cluster Profiler R package was used for Gene Ontology (GO) annotation and Kyoto Encyclopedia of Genes and Genomes (KEGG) pathway enrichment analysis of differentially expressed genes (DEGs). Specifically, adefovir and tenofovir target genes were obtained from step 2.2.1. In addition, osteoporosis-related genes were obtained from the GSE56814 dataset (28, 29).

### 2.3 Molecular docking

The PDB structure files of target proteins were retrieved from the Protein Data Bank (PDB) database^5^ (30). Using PyMOL 2.3.0, water molecules, heteroatoms and other non-critical non-protein elements were removed to ensure a clean structure of the target protein. The SDF structure files of adefovir and tenofovir were downloaded from PubChem database (see text footnote 1) (24) and then were converted to pdbqt format by AutoDockTools (v1.5.7). Autodock Vina software was used to perform molecular docking process. PyMol 2.3.0 software was used to facilitate the visualization of the docking results pertaining to the optimal conformation.

## 3 Results

### 3.1 Real-world data analysis

#### 3.1.1 Descriptive analyses

**Figure 1:**
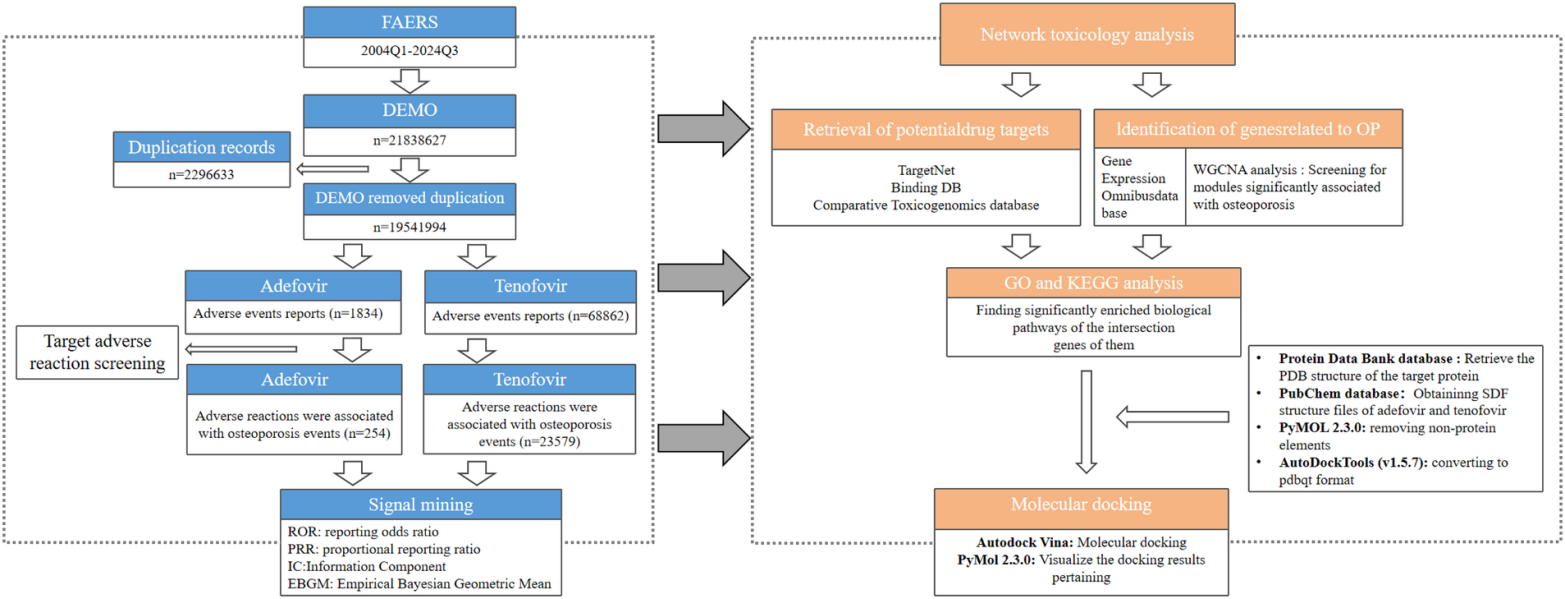
Schematic illustration of studies of adefovir and tenofovir causing adverse reactions to osteoporosis. GO, Gene Ontology; KEGG, Kyoto Encyclopedia of Genes and Genomes.

During the period of testing from Q1 2004 through Q3 2024, the FAERS database recorded 1,834 and 68,862 reports related to adverse reactions triggered by Adefovir and Tenofovir treatment of CHB, respectively (Figure 1). The basic characteristics of the patients are shown in Table 1. In the gender distribution of treatment with Adefovir, the incidence of AE was significantly higher in the male patient population (*n* = 1,154, 62.9%) than in the female patient population (*n* = 454, 24.8%). When specific to osteoporotic events, the number of male patients (*n* = 163) was approximately three times that of female patients (*n* = 65). The gender distribution of AEs induced by Tenofovir was similar to that of Adefovir (male *n* = 42,205, female *n* = 17,948). In terms of age composition, Adefovir produced adverse reactions that were prevalent in the 18–65-year-old patient population (*n* = 994, 54.2%). Tenofovir, on the other hand, had a very low incidence in patients aged 18–65 years (*n* = 70, 0.1%), and was more common in the older patient group aged 65–85 years (*n* = 36,949, 53.7%). In addition, among patients taking Adefovir or Tenofovir, compared with patients weighing less than 50 kg (Adefovir *n* = 25; Tenofovir *n* = 1,325) and more than 100 kg (Adefovir *n* = 8; Tenofovir *n* = 998), patients weighing between 50 kg and 100 kg had a larger proportion of people (Adefovir *n* = 181; Tenofovir *n* = 6,233).

**Table 1:** Basic information about adefovir and tenofovir and osteoporosis events.

Characteristics	Adefovir		Tenofovir	
	Total	SMQ-Osteoporosis	Total	SMQ-Osteoporosis
Number of events	1,834	254	68,862	23,579
Sex, *n* %				
Female	454 (24.8%)	65 (25.6%)	17,948 (26.1%)	6,330 (26.8)
Male	1,154 (62.9%)	163 (64.2%)	42,205 (61.3%)	15,621 (66.2)
Missing/unknown	226 (12.3%)	269 (10.2%)	8,709 (12.6%)	1,628 (6.9)
Age, *n* %				
<18	7 (0.4%)	0 (0%)	702 (1.0%)	23 (0.1%)
18–64.9	994 (54.2)	173 (68.1%)	70 (0.1%)	5 (0.0%)
65–85	245 (13.4%)	35 (13.8%)	36,949 (53.7%)	14,590 (61.9%)
>85	1 (0.1%)	1 (0.4%)	3,337 (4.8%)	863 (3.7%)
Missing/unknown	587 (32.0%)	45 (17.7%)	27,804 (40.4%)	8,098 (34.3%)
Weight, *n* %				
<50 kg	25 (1.4%)	3 (1.2%)	1,325 (1.9%)	73 (0.3%)
>100 kg	8 (0.4%)	0 (0%)	998 (1.4%)	361 (1.5%)
50–100 kg	181 (9.9%)	15 (5.9%)	6,233 (9.1%)	1,659 (7.0%)
Missing/unknown	1,620 (88.3%)	236 (92.9%)	60,306 (87.6%)	21,486 (91.1%)
OUTC_COD				
CA	9 (0.5%)	0 (0%)	1,444 (2.1%)	1 (0.0%)
DE	115 (6.3%)	1 (0.4%)	3,053 (4.4%)	244 (1.0%)
DS	45 (2.5%)	6 (2.4%)	665 (1.0%)	40 (0.2%)
HO	566 (30.9%)	144 (56.7%)	8,500 (12.3%)	1,231 (5.2%)
LT	20 (1.1%)	3 (1.2%)	699 (1.0%)	4 (0.0%)
OT	981 (53.5%)	99 (39.0%)	39,061 (56.7%)	18,808 (79.8%)
RI	1 (0.1%)	0 (0%)	39 (0.1%)	1 (0.0%)
Missing	97 (5.3%)	1 (0.4%)	15,401 (22.4%)	3,250 (13.8%)
OCCP_COD				
Consumer	342 (18.6%)	26 (10.2%)	14,933 (21.7%)	4,021 (17.1%)
Health professional	115 (6.3%)	14 (5.5%)	5,452 (7.9%)	69 (0.3%)
Lawyer	–	–	24,607 (35.7%)	18,438 (78.2%)
Physician	692 (37.7%)	110 (43.3%)	11,400 (16.6%)	690 (2.9%)
Other health-professional	448 (24.4%)	101 (39.8%)	6,054 (8.8%)	278 (1.2%)
Pharmacist	68 (3.7%)	1 (0.4%)	5,075 (7.4%)	64 (0.3%)
Registered Nurse	–	–	1 (0.0%)	0 (0%)
Missing	169 (9.2%)	2 (0.8%)	5,075 (7.4%)	64 (0.3%)

CA, Congenital Anomaly; DE, Death; DS, Disability; HO, Hospitalization-Initial or Prolonged; LT, Life-Threatening; OT, Other Serious Important Medical Event; RI, Required Intervention to Prevent Permanent.

#### 3.1.2 Signal detection

**Figure 2:**
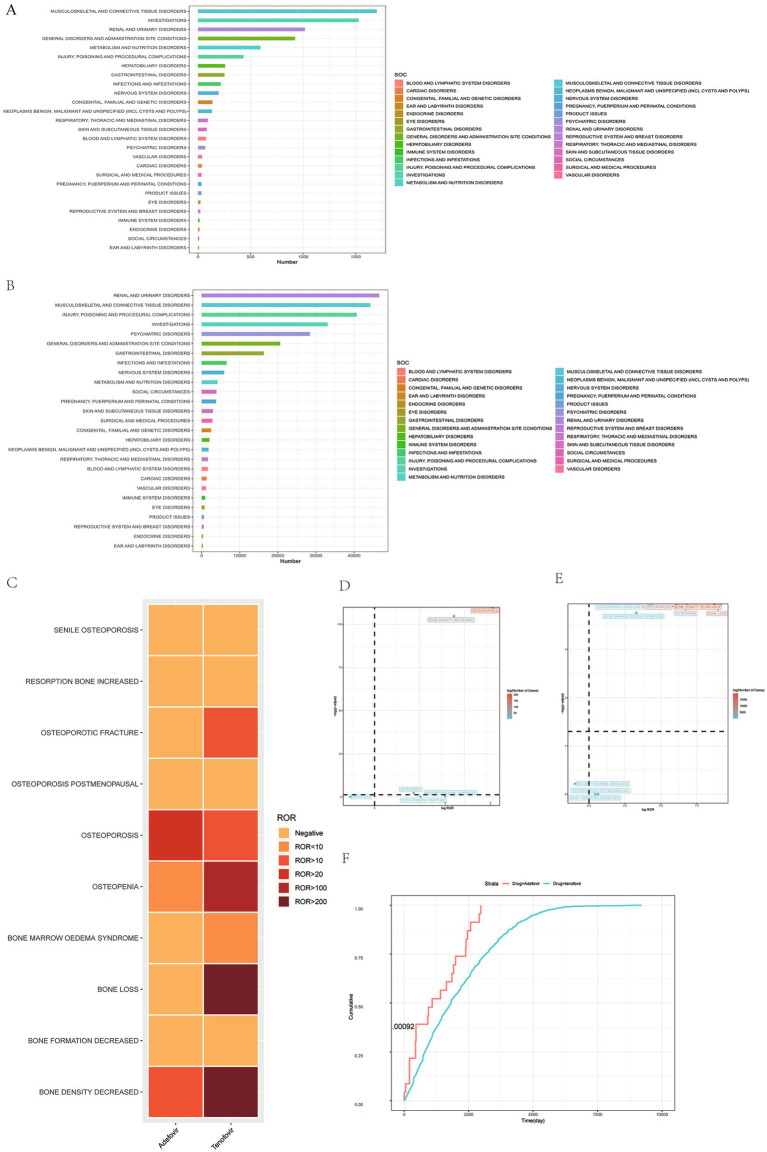
Scanning for adverse osteoporosis events associated with adefovir and tenofovir based on the FAERS database. **(A)** Bar graph showing the number of AE cases reported for each SOC level of adefovir in the FAERS database. **(B)** Bar graph showing the number of AE cases reported for tenofovir at each SOC level in the FAERS database. **(C)** Heatmap showing the ROR of 10 osteoporosis adverse events in the FAERS database under different NAs treatment strategies including adefovir, tenofovir. **(D)** Risk signal volcano map of adefovir in the North American population. **(E)** Risk signal volcano plot for tenofovir in the North American population. **(F)** Adverse event induction time plots for adefovir and tenofovir. NAs, nucleoside/nucleotide analogs; FAERS, Food and Drug Administration (FDA) Adverse Event Reporting System (FAERS); AE, adverse event; SOC, systemic organ classification; ROR, reporting odds ratio; CI, confidence interval.

According to the statistics, a total of 27 organ systems were affected by Adefovir and Tenofovir-related adverse events at the SOC level. The system that was accrued the most was musculoskeletal and connective tissue disorders (*n* = 45,997), while the SOC with the lowest number of accrued SOCs was congenital, familial and genetic disorders (*n* = 2,620). In terms of signal intensity, renal and urinary disorders showed the strongest positive signals in both drugs, especially in Tenofovir (ROR = 11.23, 95% CI = 11.12–11.34) (Figures 2A,B).

When focusing on osteoporosis-related adverse events caused by Adefovir and Tenofovir, the presence of positive signals was retrieved for both (Figure 2C). Of these, only two positive PTs were retrieved in Adefovir-associated osteoporosis-associated PTs, namely osteoporosis (ROR = 38.42, 95% CI = 33.46–44.13, *p* = 0) and bone density decreased (ROR = 11.56, 95% CI = 8.78–15.23, *p* = 7.21E−10^3^) (Figure 2D). Secondly, the highest number of osteoporotic events complicated by Tenofovir administration (*n* = 45,216) contained five positive PTs. Bone loss (ROR = 579.00, 95%CI = 557.46–601.37, p = 0) was the strongest positive signal intensity for an osteoporosis adverse reaction. In addition, the highest number of occurrences was bone density decreased (*n* = 19,916), which was also retrieved with a high positive signal. (ROR = 423.92, 95%CI = 413.63–434.46, p = 0) (Figure 2E).

In addition, SMQ further improves the consistency and comparability of the data by having a more rigorous screening and integration mechanism to group multiple PTs with similar presentations or common pathological pathways. Studies based on the SMQ level showed that strong positive signals were detected for both Adefovir and Tenofovir, and the positive signal value for Tenofovir (ROR = 197.88,95% CI = 195.28–200.52) was much greater than that for Adefovir (ROR = 18.96, 95% CI = 16.78–21.42).

Subsequently, we further explored the potential association between Adefovir and Tenofovir induced hair osteoporosis risk in different populations. There were differences in the results of Adefovir versus Tenofovir in different gender populations. The results of SMQ levels showed that in Adefovir, osteoporosis-related adverse events in the female group (female SMQ ROR = 21.49, 95%CI = 16.84–27.43, *p* < 0.05) presented a stronger signal intensity than in the male group (male SMQ ROR = 14.81, 95%CI = 12.73–17.22, *p* < 0.05). In contrast, the overall positive signal value for osteoporosis adverse events in women was much lower than the overall positive signal value in men in those treated with Tenofovir. The intensity of positive signals for adverse events in the male group (male SMQ ROR = 349.27, 95%CI = 341.33–357.4, *p* < 0.05) was approximately two times higher than in the female group (female SMQ ROR = 164.63 95%CI = 161.14–168.2, *p* < 0.05).

**Figure 3:**
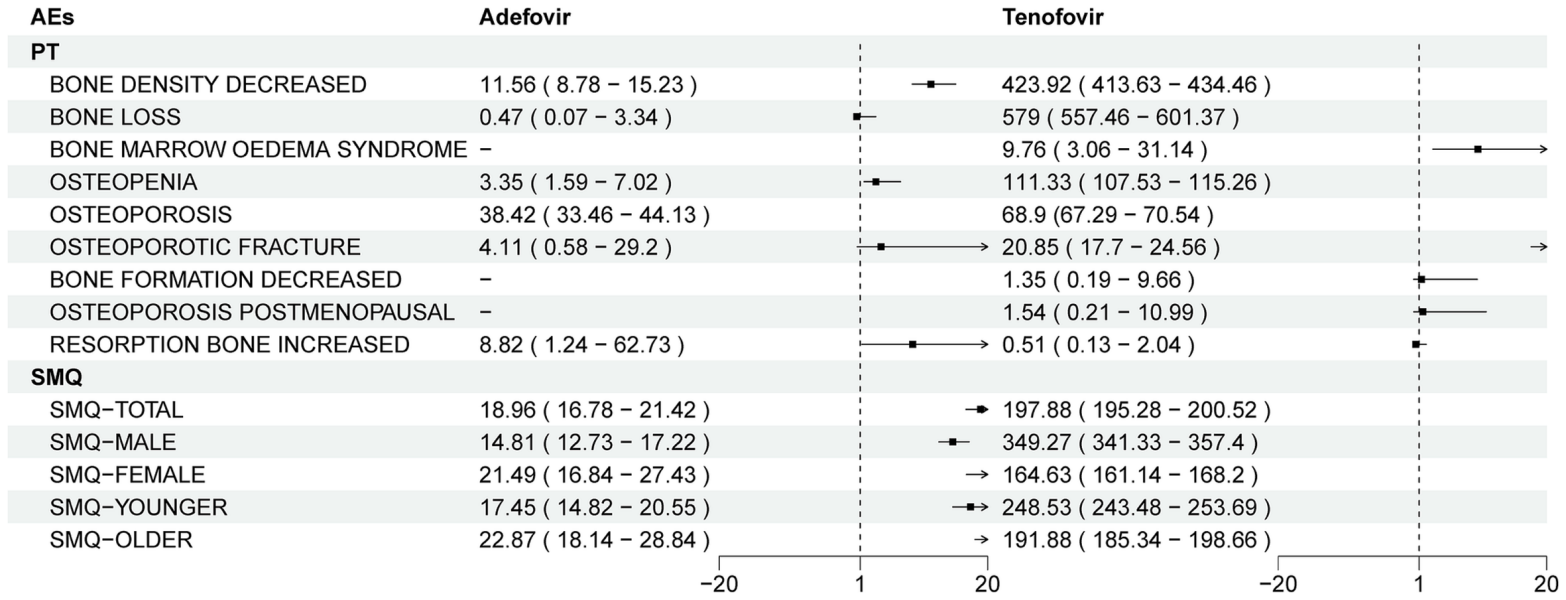
Differential risk signal analyses for adefovir and tenofovir at the PT and SMQ levels, respectively. PTs, preferred term; SMQ, Standardized MedDRA Queries.

In addition, the study was divided into two cohorts, low (<60 years) and high (≥60 years), for age subgroup analysis. At the SMQ level, the results in Adefovir were opposite to those in Tenofovir. In Adefovir, stronger positive signals were detected in the higher age group (SMQ ROR = 22.87, 95%CI = 18.14–28.84, *p* < 0.05) than in the lower age group (SMQ ROR = 17.45, 95%CI = 14.82–20.55, *p* < 0.05) (Figure 3; Table 2).

**Table 2:** Subgroup analysis of adefovir and tenofovir based on age and gender.

Drug	Category	AEs	N	ROR (95%Cl)	PRR (χ^2^)	EBGM (EBGM05)	IC (IC025)	*p* value
Adefovir	PT	Bone density decreased	51	11.56 (8.78–15.23)	11.5 (488.22)	11.48 (9.12)	3.52 (3.12)	<0.001
		Bone loss	1	0.47 (0.07–3.34)	0.47 (0.59)	0.47 (0.09)	−1.09 (−3.13)	>0.99
		Bone marrow oedema syndrome	-	-	-	-	-	-
		Osteopenia	7	3.35 (1.59–7.02)	3.34 (11.5)	3.34 (1.8)	1.74 (0.72)	>0.99
		Osteoporosis	207	38.42 (33.46–44.13)	37.45 (7,308.06)	37.25 (33.17)	5.22 (5.02)	<0.001
		Osteoporotic fracture	1	4.11 (0.58–29.2)	4.11 (2.35)	4.11 (0.8)	2.04 (0)	>0.99
		Bone formation decreased	-	-	-	-	-	-
		Osteoporosis postmenopausal	-	-	-	-	-	-
		Resorption bone increased	1	8.82 (1.24–62.73)	8.82 (6.93)	8.81 (1.71)	3.14 (1.1)	>0.99
	SMQ	SMQ − TOTAL	268	18.96 (16.78–21.42)	18.35 (4,393.38)	18.31 (16.53)	4.19 (4.02)	<0.001
		SMQ − MALE	175	14.81 (12.73–17.22)	14.36 (2,171.41)	14.31 (12.61)	3.84 (3.62)	<0.001
		SMQ − FEMALE	67	21.49 (16.84–27.43)	20.74 (16.91)	4.37 (4.02)	20.77 (1,261.24)	<0.001
		SMQ − YOUNGER	67	21.49 (16.84–27.43)	20.74 (16.91)	4.37 (4.02)	20.77 (1,261.24)	<0.001
		SMQ − OLDER	74	22.87 (18.14–28.84)	22.2 (1,494.95)	22.13 (18.22)	4.47 (4.13)	<0.001
Tenofovir	PT	Bone density decreased	19,916	423.92 (413.63–434.46)	393.37 (2,557,866.25)	129.69 (127.05)	7.02 (6.99)	<0.001
		Bone loss	10,533	579 (557.46–601.37)	556.91 (1,498,769.8)	143.51 (139.03)	7.17 (7.13)	<0.001
		Bone marrow oedema syndrome	3	9.76 (3.06–31.14)	9.76 (22.45)	3.22 (1.74)	9.34 (3.54)	0.59
		Osteopenia	5,066	111.33 (107.53–115.26)	109.3 (346,508.18)	70.01 (68.01)	6.13 (6.08)	<0.001
		Osteoporosis	9,542	68.9 (67.29–70.54)	66.55 (457,793.55)	49.67 (48.7)	5.63 (5.6)	<0.001
		Osteoporotic fracture	159	20.85 (17.7–24.56)	20.84 (2,708.72)	18.89 (16.48)	4.24 (4)	<0.001
		Bone formation decreased	1	1.35 (0.19–9.66)	1.35 (0.09)	0.43 (−1.62)	1.35 (0.26)	>0.99
		Osteoporosis postmenopausal	1	1.54 (0.21–10.99)	1.54 (0.19)	0.62 (−1.44)	1.53 (0.3)	>0.99
		Resorption bone increased	2	0.51 (0.13–2.04)	0.51 (0.95)	−0.97 (−2.64)	0.51 (0.16)	>0.99
	SMQ	SMQ − TOTAL	45,223	197.88 (195.28–200.52)	165.59 (3,979,447.71)	89.37 (88.38)	6.48 (6.46)	<0.001
		SMQ − MALE	30,091	349.27 (341.33–357.4)	291.21 (2,211,339.52)	74.58 (73.15)	6.22 (6.2)	<0.001
		SMQ − FEMALE	12,938	164.63 (161.14–168.2)	136.31 (1,298,795.73)	101.96 (100.14)	6.67 (6.64)	<0.001
		SMQ − YOUNGER	26,387	248.53 (243.48–253.69)	206.45 (2,009,876.54)	77.37 (76.05)	6.27 (6.25)	<0.001
		SMQ − OLDER	4,778	191.88 (185.34–198.66)	159.01 (582,776.21)	123.57 (120.04)	6.95 (6.9)	<0.001

ROR, Report Odds Ratio; PRR, Proportional Reporting Ratio; IC, Information Component; EBGM, Empirical Bayesian Geometric Mean; *p* value, Adjusted *p* value.

#### 3.1.3 Time-to-onset analysis

In an assessment to evaluate the time to osteoporotic events after Adefovir versus Tenofovir treatment, the mean time to induction for Adefovir was 1,302.48 ± 1,001.47 days and the median time to induction was 1,089 days. The time to induction subgroup showed that approximately 78.26% of Adefovir users experienced osteoporosis adverse events after 1 year. The mean induction time for Tenofovir was 2,123.37 ± 1,567.41 with a median induction time of 1,814 days. As with Adefovir, the majority of its adverse events occurred after 1 year (approximately 90.31%). In addition, we analyzed the time of onset of osteoporosis adverse events associated with both drugs. The results showed a statistically significant difference in the induction time between Adefovir and Tenofovir (*p* = 0.00092) (Figure 2F). Weibull distribution modeling studies have been used to determine whether the risk of drug-related AE exhibits a time trend. Of these, osteoporosis was mainly randomized after Adefovir treatment and showed a random failure curve. Tenofovir, on the other hand, exhibited a wear failure curve, which suggests that Tenofovir-induced osteoporosis adverse events progressively increase with duration of dosing (Table 3).

**Table 3:** Weibull shape parameter test for adefovir and tenofovir.

Drug	Average (d) ± SD	Median (d)	Scale parameter: *α* (95% CI)	Shape parameter: *β* (95% CI)	Type
Adefovir	1,302.48 ± 1,001.47	1,089	1,331.68 (801.73–1,861.63)	1.07 (0.70–1.44)	Random failure
tenofovir	2,123.37 ± 1,567.41	1,814	2,287.95 (2,238.97–2,336.93)	1.29 (1.26–1.32)	Wear failure

### 3.2 Network toxicology

#### 3.2.1 Identification of genes related to osteoporosis

**Figure 4:**
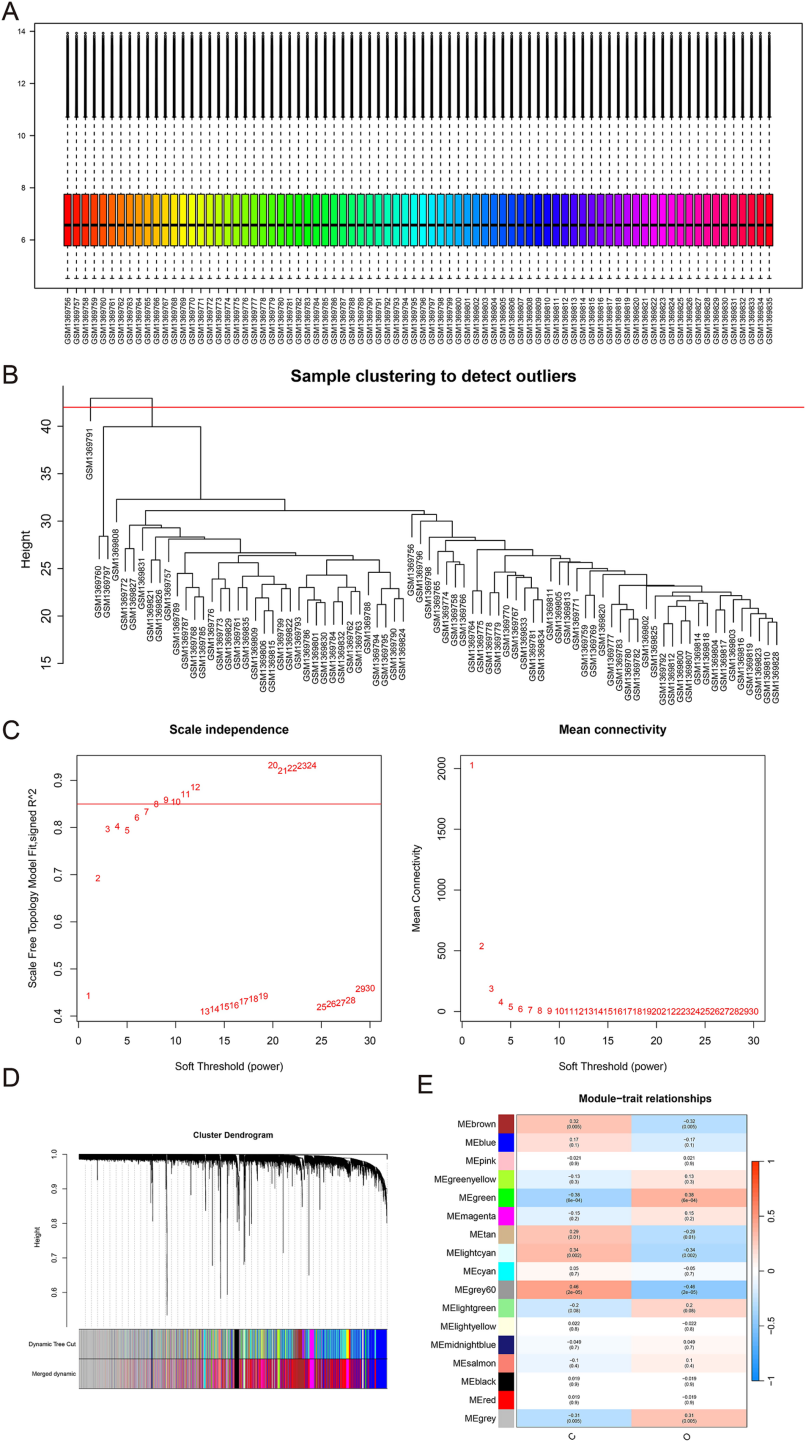
**(A)** Box plots of raw data normalized between samples. **(B)** Sample clustering to detect outliers. **(C)** Determination of the optimal soft threshold. **(D)** Dendrogram illustrating hierarchical clustering of genes based on their modular characteristics. **(E)** Correlation analysis between gene modules and clinical traits. The color intensity in the image denotes the strength and direction of the correlation between gene modules and clinical traits: red signifies a positive correlation, blue indicates a negative correlation, and deeper colors reflect stronger correlations. A lower *p*-value suggests a higher level of significance.

GSE56814 dataset, which included 40 women with high bone density and 40 women with low bone density, was downloaded from GEO database and analyzed in this study. Normalization was performed and visualized in Figure 4A. WGCNA was applied to identify the modules significantly associated with osteoporosis. GSM1369791 was identified as abnormal sample and was removed (Figure 4B). A soft threshold power of *β* = 9 (scale-free R2 = 0.858) was selected (Figure 4C). As shown in Figures 4D,E, 17 co-expression modules were identified, of which the brown module, green module, tan module, gray 60 module, and lightgreen module were significantly associated with osteoporosis. Combine genes in these modules, a total of 1,473 genes were identified as genes closely associated with osteoporosis.

#### 3.2.2 Identification of genes related to drugs influencing osteoporosis

**Figure 5:**
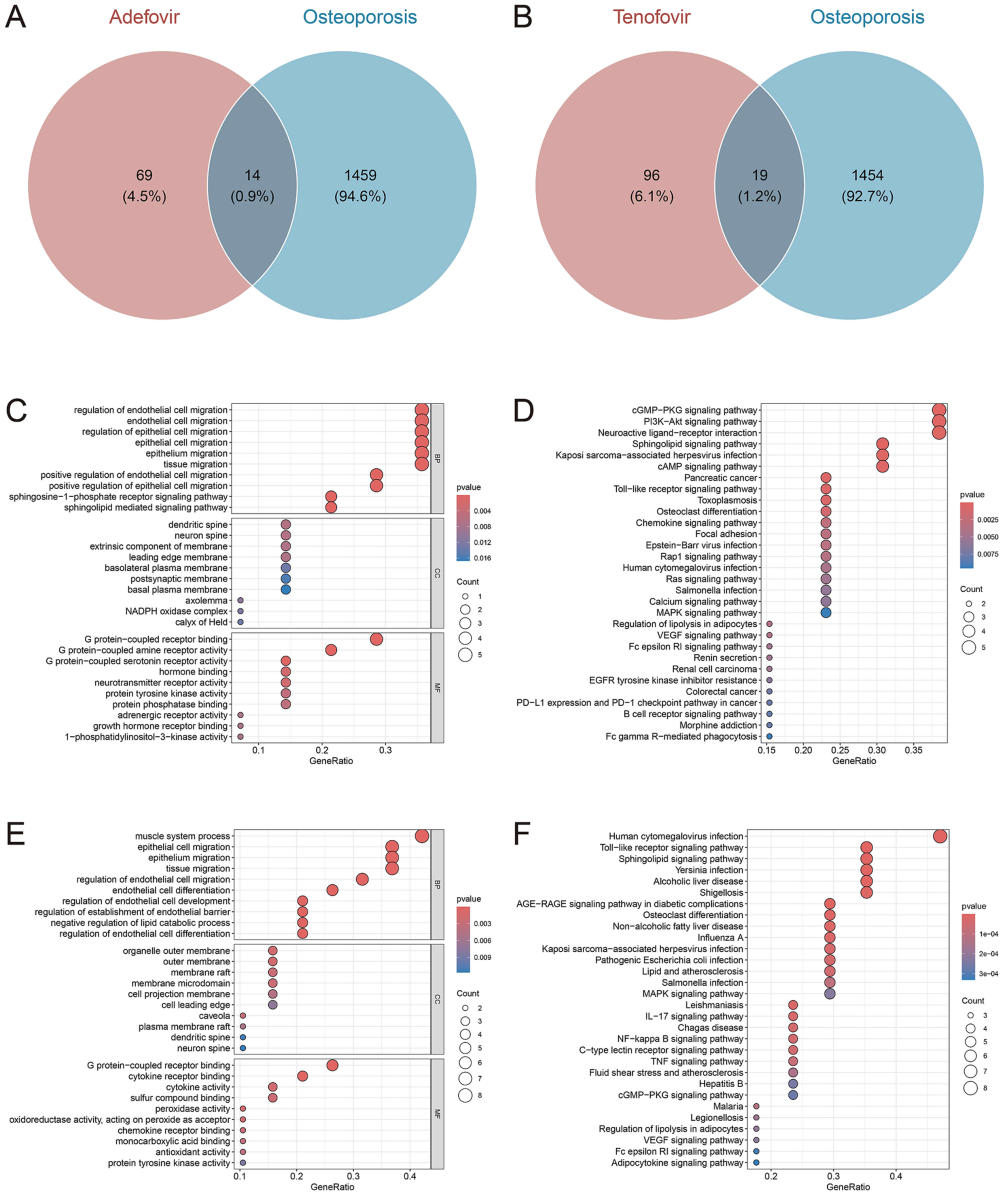
Venn diagrams of gene targets for adefovir **(A)**, and tenofovir **(B)** influencing osteoporosis. The GO **(C)** and KEGG **(D)** plots of the 14 gene targets for adefovir influencing osteoporosis. The GO **(E)** and KEGG **(F)** plots of the 19 gene targets for tenofovir influencing osteoporosis.

Synthesizing Binding DB, CTD, and TargetNet, a total of 83 genes were identified as the potential targets of adefovir (Supplementary Table 2), 115 genes were identified as the potential targets of tenofovir (Supplementary Table 3). By intersecting these gene targets with 1,473 genes closely associated with osteoporosis, 14 overlapping genes were identified as potential gene targets for adefovir influencing osteoporosis (Figure 5A), and 19 overlapping genes were selected as potential gene targets for tenofovir influencing osteoporosis (Figure 5B). For adefovir, GO enrichment analysis (Figure 5C) suggested that cell migration, sphingosine-1-phosphate receptor signaling pathway, sphingolipid mediated signaling pathway, G protein-coupled receptor binding, G protein-coupled amine receptor activity and so on were significantly enriched. KEGG enrichment analysis indicated the significant enrichment of cGMP-PKG signaling pathway, PI3K-Akt signaling pathway, Toll-like receptor signaling pathway and so on (Figure 5D). For tenofovir, GO enrichment analysis revealed that migration of epithelial cell and endothelial cell, differentiation of endothelial cell, muscle system process, G protein-coupled receptor binding, and cytokine activity, and so on were significantly enriched (Figure 5E). KEGG enrichment analysis suggested that Toll-like receptor signaling pathway, Sphingolipid signaling pathway, MAPK signaling pathway, and IL-17 signaling pathway, and so on were significantly enriched (Figure 5F).

### 3.3 Molecular docking

**Figure 6:**
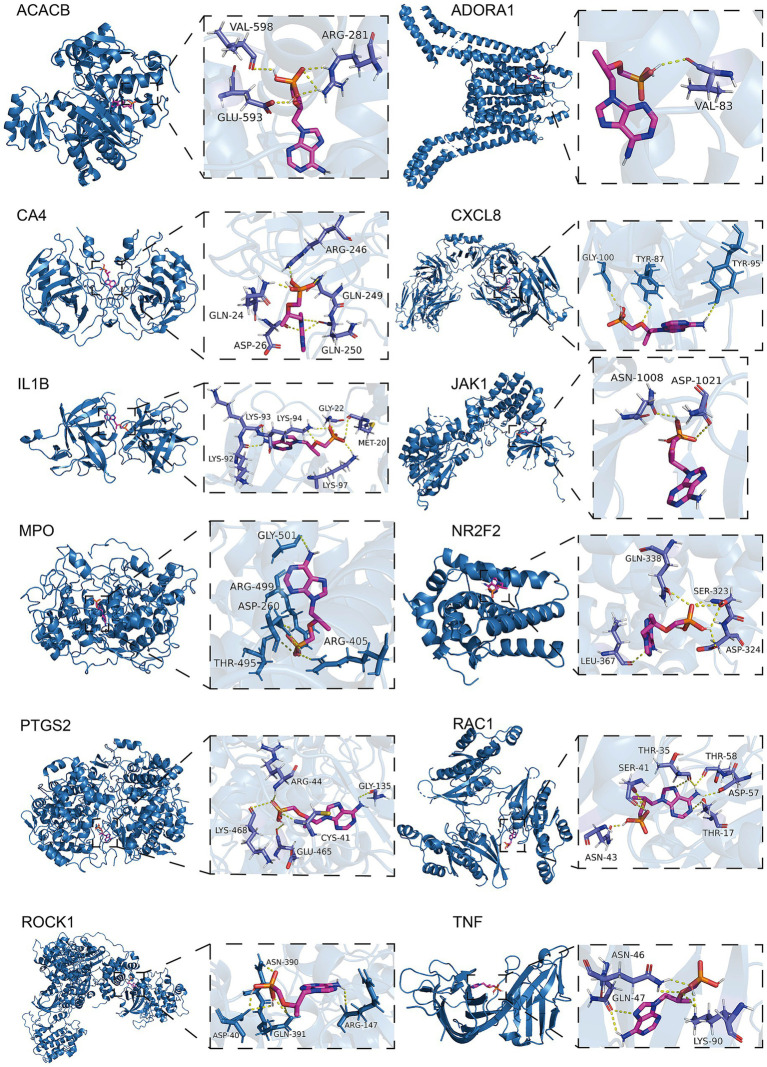
Molecular docking analyses of adefovir and ADORA1, HTR5A, JAK1, LCN2, NR2F2, PDE3A, PIK3CG, and RAC1.

**Figure 7:**
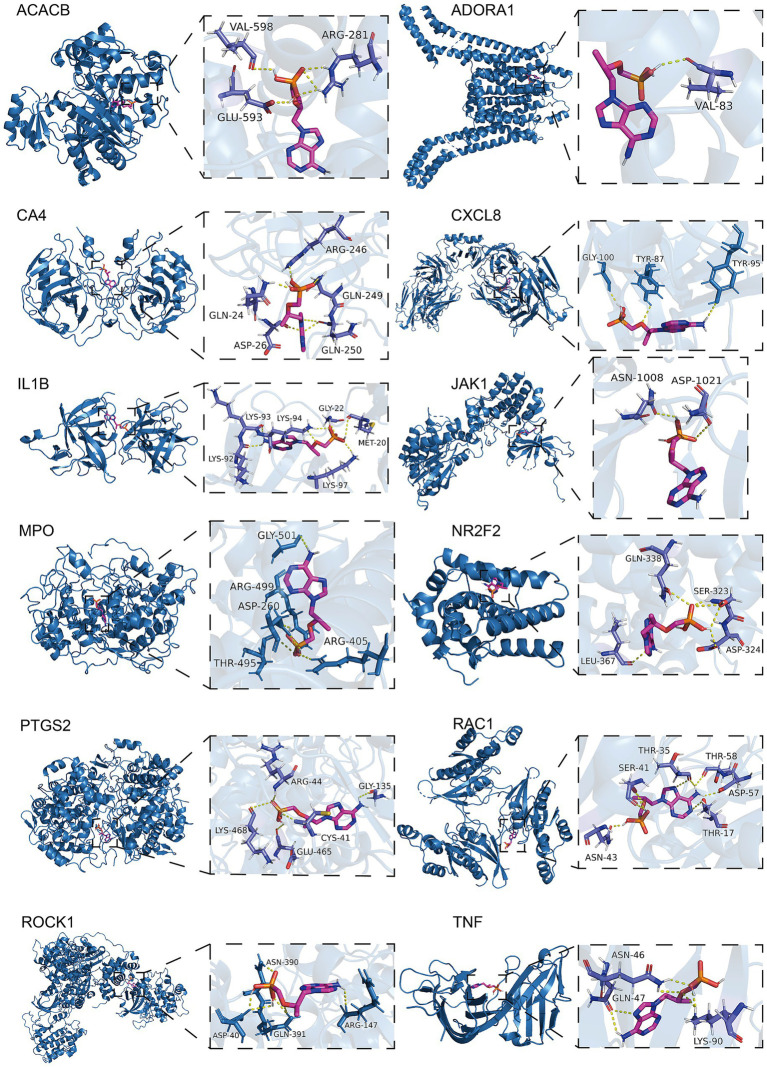
Molecular docking analyses of tenofovir and ACACB, ADORA1, CA4, CXCL8, IL1B, JAK1, MPO, NR2F2, PTGS2, RAC1, ROCK1, and TNF.

Using molecular docking methods, we further assessed the interaction between adefovir, tenofovir and gene targets of them influencing osteoporosis. As shown in Figure 6, there was a good combination ability between adefovir and ADORA1, HTR5A, JAK1, LCN2, NR2F2, PDE3A, PIK3CG, and RAC1. As shown in Figure 7, the combination between tenofovir and ACACB, ADORA1, CA4, CXCL8, IL1B, JAK1, MPO, NR2F2, PTGS2, RAC1, ROCK1, and TNF was excellent. The detailed information about the binding energies was shown in Table 4.

**Table 4:** Results of molecular docking.

Drug	Targets	PDB ID	Binding affinity (kcal/mol)
Adefovir	ADORA1	5UEN	−5.6
	HTR5A	7UM4	−6.3
	JAK1	4E5W	−6.6
	LCN2	3S26	−5.8
	NR2F2	3CJW	−5.8
	PDE3A	7L27	−6.5
	PIK3CG	6AUD	−6.5
	RAC1	2NZ8	−6.3
Tenofovir	ACACB	3GLK	−6.5
	ADORA1	5UEN	−6
	CA4	3FW3	−6.5
	CXCL8	6WZM	−5.9
	IL1B	8C3U	−7
	JAK1	4E5W	−7.1
	MPO	5MFA	−8.2
	NR2F2	3CJW	−5.9
	PTGS2	5F19	−7.5
	RAC1	2P2L	−6.9
	ROCK1	3V8S	−6.9
	TNF	5M2J	−6.2

## 4 Discussion

HBV is one of the most important causes of liver disease and poses a major threat to global public health. Currently, NAs drug therapy such as adefovir and tenofovir are recommended as first-line HBV regimens and are widely used in clinical practice (11, 12). However, there is no conclusive information about the effects of these two drugs on bone mineral density (BMD) (31, 32). Therefore, this study confirmed the association between adefovir and tenofovir and osteoporotic events through real-world feedback. This confirmation was based on the assessment of overall SMQ and PT level signaling. In addition, we delved into the specific associations of these drugs through further network toxicology and molecular docking studies.

**Figure 8:**
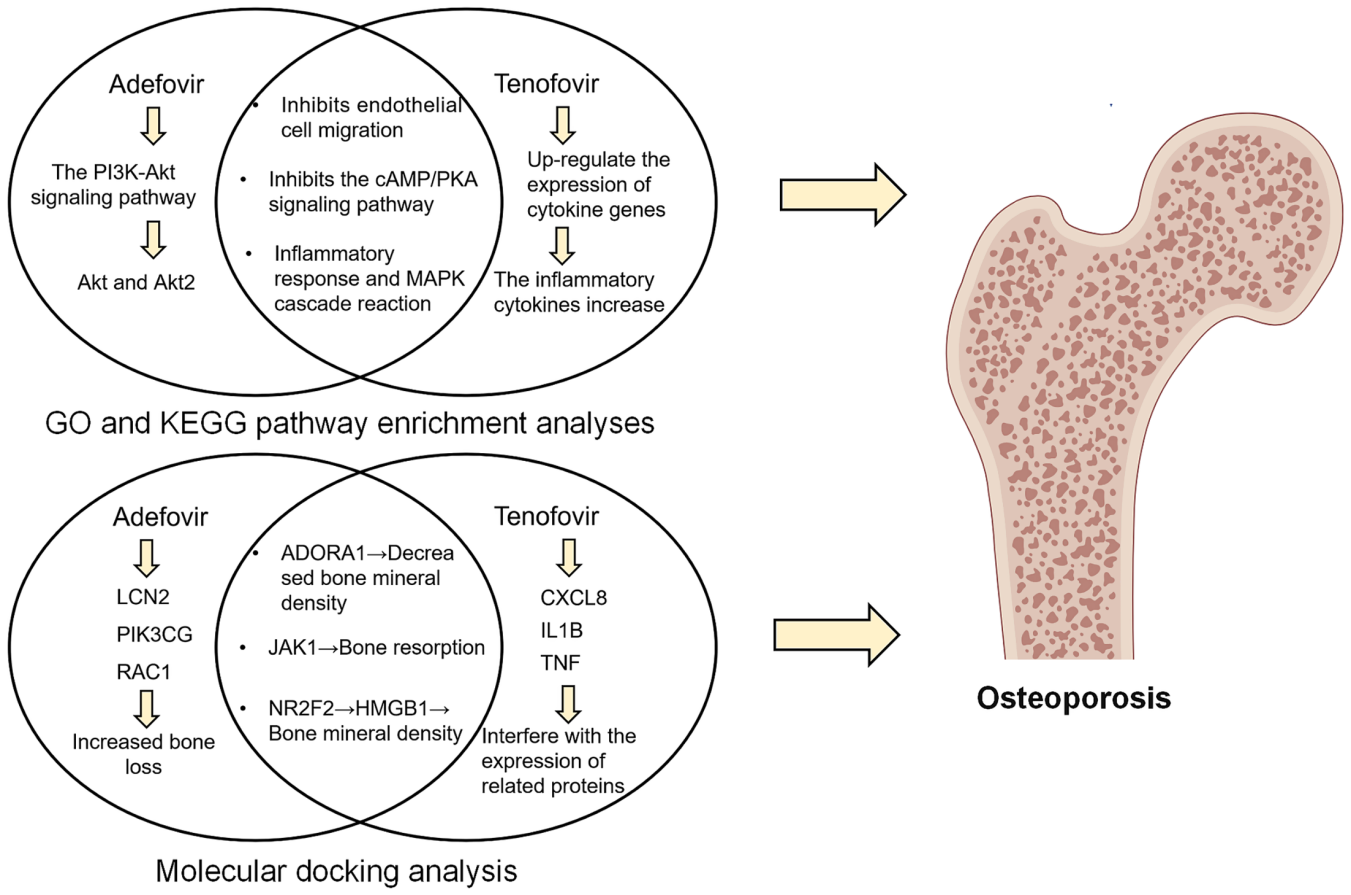
Schematic diagram of the molecular mechanism of GO and KEGG pathway enrichment analysis and molecular docking analysis.

Specifically, in the present study, there was a signal for skeletal adverse events at the PT level for both adefovir and tenofovir. Subgroup analyses showed that adefovir was more likely to cause osteoporosis in older adults and women, while tenofovir showed the opposite trend. In addition, GO and KEGG analyses showed that both drugs may jointly promote osteoporosis through pathways such as cell migration, G protein-coupled receptor and Toll-like receptor signaling pathways (Figure 8). The latest study further proves our point. Regarding adefovir, several case reports have indicated that patients taking adefovir are prone to bone problems such as osteochondrosis. In addition, patients’ skeletal conditions improved significantly after discontinuing adefovir (33, 34). Also, tenofovir has been found to increase the risk of osteoporosis, which was confirmed by continuous monitoring of bone mineral density in patients with chronic hepatitis B treated with tenofovir, which showed a significant decrease in the tenofovir group from the mean percentage of baseline throughout the course of treatment (35).

Notably, our subgroup analysis of different populations suggests that the bone effects from adefovir and tenofovir accrue to different populations. Adefovir was more likely to affect the elderly and women. Tenofovir, on the contrary, often induces osteoporosis in young people and men. The specific metabolism of the two drugs may play a key role in this particular phenomenon. Adefovir is mainly excreted in its native form via the kidneys, a relatively simple metabolic process that makes renal function crucial for its excretion (36). In the elderly, renal function tends to decline significantly due to reduced renal units, vascular aging, and dysregulation of the renin-angiotensin-aldosterone system, which results in the inability to metabolize adefovir properly, leading to a higher signal for adverse events of bone loss (37). In addition, the elderly population is often associated with multiple chronic diseases such as diabetes mellitus, implying that further impairment of renal function in the elderly due to factors such as high glucose provides a synergistic effect on bone loss due to adefovir (38). Similarly, in the female population, the effects of adefovir on bone mass are also exacerbated by reduced renal blood flow due to decreased ovarian function and estrogen levels during menopause (39). Unlike adefovir, tenofovir has a more complex metabolic pattern, and it has even been able to rescue renal impairment caused by other NAs (40). This causes it to accrue to a population with completely different characteristics than adefovir. A retrospective study from Hong Kong, China suggests that men are more likely to experience osteoporosis and fractures after tenofovir use (41). In addition, in a study of a Human Immunodeficiency Virus (HIV)-negative population using tenofovir, men also showed a predisposition to reduced bone density (42). Furthermore, tenofovir has been observed to exhibit a greater capacity to regulate cytokine and ion levels in comparison to adefovir (43). Furthermore, hormonal secretion regulation mechanisms, such as parathyroid hormone, exhibit heightened sensitivity in younger populations (44). Consequently, when Tenofovir leads to the downregulation of calcium ions and other levels in the body, the younger organism will activate the release of bone calcium levels more quickly than the older group, which will lead to the occurrence of osteoporosis more easily.

Time-series analysis suggested that Adefovir caused osteoporosis to exhibit random failure curve, suggesting that osteoporosis symptoms persisted over time (45). Tenofovir, on the other hand, exhibits a wear failure curve, implying that osteoporosis adverse events progressively increase with Tenofovir dosing time (46). This suggests that long-term use of these two drugs should be avoided as much as possible in the clinic, and when they have to be used, they should be used prophylactically as early as possible with anti-osteoporotic drugs.

GO analysis suggested that cell migration and G-protein-coupled receptor binding are shared pathways by which adefovir and tenofovir trigger osteoporosis. This suggests that adefovir and tenofovir may inhibit endothelial cell migration and the process of neovascularization through mechanisms such as disrupting endothelial cell microfilament structure and producing endothelial cytotoxicity (47, 48). This inhibition leads to a decrease in the production of vascular endothelial growth factor, which decreases the number of cells and new blood vessels reaching the bone-forming region, ultimately resulting in a paucity of blood supply to the bone tissue and a slowing of bone-forming activity (48). In addition, mitochondria play an important role in cell migration. Adefovir and tenofovir induce mitochondrial damage by inhibiting the mitochondrial chaperone TRAP1 and the mtDNA replication protein SSBP1, thereby affecting the energy supply required for cell migration. This cascade of events further impairs osteoblast activity during bone formation, ultimately promoting osteoporosis (49, 50).

Furthermore, upregulation of G protein-coupled receptor binding (GPCR) is another factor in the induction of osteoporosis by adefovir and tenofovir. Among them, 5-hydroxy tryptamine (5-HT) receptor as a GPCR was shown to be widely present in osteoclasts and osteoblasts (51). Up-regulation of 5-HT receptor inhibits the cAMP/PKA pathway, and inhibition of PKA leads to phosphorylation of activating transcription factor 4 (ATF4), which stimulates osteoclast differentiation and causes a decrease in bone density (52). More in-depth studies of pathway mechanisms have shown that both adefovir and tenofovir have been found to exert their effects on bone density through the Toll-like receptor signaling pathway. The Toll-like receptor (TLR) signaling pathway is an important pathway for the activation of immune responses, and the majority of TLRs use a MyD88-dependent pathway to activate the transcription factors NF-κB and protein kinase to induce inflammatory cytokine release (53). Among them, the inflammatory response and mitogen activated protein kinase (MAPK) cascade can promote osteoclast activity, which in turn leads to an imbalance in bone resorption and bone formation, an important causative factor in osteoporosis (54).

In addition to the common pathway, adefovir regulates cell proliferation and differentiation by affecting the PI3K-Akt signaling pathway. PI3K activation recruits the downstream signaling molecule protein kinase B (AKT), which promotes mammalian target of rapamycin (mTOR) activation, affects osteoblast differentiation and inhibits apoptosis, improves osteoblast survival (55). Whereas, the nucleotide analog Adefovir cellular metabolite can inhibit normal bone formation function by binding to Akt proteins and blocking their movement to the cell membrane and phosphorylation (56). In addition to this, deletion of Akt2, another isoform of AKT, has also been found to decrease the bone resorption capacity of osteoclasts (57). Therefore, when this pathway is upregulated, it increases osteoclast activity and further develops osteoporosis.

Notably, unlike adefovir, tenofovir additionally contributes to the development of osteoporosis by affecting inflammatory cytokine pathways such as IL-17, IL-1β, and TNF. A study on the effects of tenofovir on the mucosal tissue environment likewise found that tenofovir upregulates cytokine gene expression in epithelial cells and fibroblasts, which can increase the level of secretion of inflammatory factors such as tumor necrosis factor-alpha (TNF-α) and IL-8, which in turn can have some negative effects on bone health (58, 59). Further studies found that IL-17 can promote the expression of matrix metalloproteinase-9 (MMP-9) in osteoblasts and enhance their ability to degrade bone matrix (60). In addition, IL-1β and TNF-α can also regulate the number of osteoblasts by up-regulating Fas-mediated apoptosis of osteoblasts, which results in an inhibitory effect on the bone formation process (61).

Molecular docking further suggested that adefovir and tenofovir, respectively, could promote the development of osteoporosis by binding to different proteins. Both adefovir and adefovir bind to ADORA1, JAK1, and NR2F2. In addition, adefovir binds well to proteins such as LCN2, PIK3CG, and RAC1, while tenofovir has good binding ability to CXCL8, IL1B, and TNF. The specific mechanisms of some of these related proteins have been elucidated. Adora1 acts as an adenosine receptor and when it is significantly up-regulated, mouse bone density is significantly reduced (62). As a key target, it binds to Adefovir and Tenofovir to promote the development of osteoporosis. Furthermore, when assessing the BMD profile of de-ovulated rats, JAK1 was found to promote bone resorption by co-activation with STAT3 (63). The link between NR2F2 and bone density is unclear, but some studies suggest that its downstream HMGB1 protein may be its core protein affecting bone density (64, 65). In addition to this, adefovir and tenofovir are able to bind specific proteins to act. For example, LCN2 was found to be reduced after laparoscopic sleeve gastrectomy (LSG) in obese Chinese women, and hormonally reduced bone density in the patients (66). Adefovir causes increased bone loss in patients by binding to LCN2, while CXCL8, a specific target of tenofovir, interferes with the expression of CXLC8, and patients ultimately experience adverse events of osteoporosis (67). Clarifying the specific protein targets of adefovir and tenofovir-induced osteoporosis will help provide more detailed recommendations for future clinical improvement of the drugs or prevention of adverse events.

Our study has the following strengths, first, we confirmed the presence of osteoporotic adverse events with adefovir and tenofovir treatment using real-world adverse event data. In addition, GO and KEGG pathway enrichment analyses clarified the biological processes and pathways that play a key role in this. Supplementarily, molecular docking techniques were used to further explore specific protein targets and direct binding processes.

However, this study still has some limitations. First, the FAERS database suffers from many selection biases and contains inaccurate or incomplete information, so we need to conduct more rigorous prospective studies to obtain a more comprehensive and accurate view. Additionally, although relevant targets and pathways can be screened by GO and KEGG pathway enrichment analysis methods, the specific mechanisms of action of adefovir and tenofovir on these signaling pathways have not been sufficiently investigated, and more experimental evidence is needed to support them in the future. Finally, the molecular docking-based approach has the disadvantage of failing to predict target up-and downregulation, which is detrimental to accurately understanding the mechanism by which chemical components act on disease targets.

## 5 Conclusion

Our study reveals the effects of Adefovir and Tenofovir on bone health from a real-world perspective and, through further analysis, identifies their core active ingredients and key target pathways that lead to OP. The aim is to provide a theoretical basis for the rational clinical use of anti-hepatitis virus drugs and the prevention and management of associated bone health risks. To provide more detailed recommendations for future clinical improvement of nucleotide analogs or prevention of their adverse events.

## Data Availability

The original contributions presented in the study are included in the article/Supplementary material, further inquiries can be directed to the corresponding author.

## Footnotes

^1^ https://pubchem.ncbi.nlm.nih.gov/

^2^ https://www.bindingdb.org

^3^ http://ctdbase.org/

^4^ http://targetnet.scbdd.com/home/index/

^5^ https://www.rcsb.org/

## Glossary

5-HT: 5-hydroxy tryptamine
AEs: Adverse events
AKT: Protein kinase B
ATF4: Activating transcription factor 4
BMD: Bone mineral density
CaSR: Calcium-sensitive receptor
CHB: Chronic hepatitis B
DEMO: Demographic information
DRUG: Drug Information
EBGM: Empirical Bayesian Geometric Mean
FAERS: FDA Adverse Event Reporting System
GEO: Gene Expression Omnibus
GPCR: G protein-coupled receptor binding
HBV: Hepatitis B virus
HCC: Hepatocellular carcinoma
HIV: Human Immunodeficiency Virus
IC: Information Component
ICSRs: Individual Case Safety Reports
INDI: Indications for Medication Administration
LSG: Laparoscopic sleeve gastrectomy
MAPK: Mitogen-activated protein kinase
MedDRA: Medical Dictionary for Regulatory Activities
MMP-9: Matrix metalloproteinase-9
mTOR: Mammalian target of rapamycin
NAs: Nucleotide and nucleoside analogs
OUTC: Patient Outcomes
PDB: Protein Data Bank
PRR: Proportional Reporting Ratio
PT: Preferred Terminology
REAC: Adverse Event Codes
ROR: Reporting Odds Ratio
RPSR: Reporting Sources
SMQs: Standardized MedDRA Queries
THER: Treatment Start and End Dates Associated with Reported Drugs
TLR: Toll-like receptor
TNF-α: Tumor necrosis factor-alpha
WGCNA: Weighted Gene Coexpression Network Analysis
