# Supplementary material for: An in-depth investigation of NAs-induced osteoporosis adverse events: a real-world, network toxicology and molecular docking analysis
Source: Front Med (Lausanne). 2025 Jul 4;12:1605024. doi: 10.3389/fmed.2025.1605024 (PMC12271188; doi:10.3389/fmed.2025.1605024)
Supplement: Supplementary file 1 [file Table_1.DOCX]

**Table S1** Four major algorithms used for signal detection

| Name of Algorithm | Formula | Positive Signal Criteria |
| --- | --- | --- |
| ROR | ROR=ad/bc | Lower Limit of 95%CI>1, N≥3 |
|  | 95%CI=e ^ln(ROR)±1.96(1/a+1/b+1/c+1/d)^0.5^ |  |
| PRR | PRR=(a(c+d))/(c(a+b)) | PRR≥2, X²≥4, N≥3 |
|  | X²=[(ad-bc)^2](a+b+c+d)/[(a+b)(c+d)(a+c)(b+d)] |  |
| BCPNN | IC=log_2_a(a+b+c+d)(a+c)(a+b) | IC_025_>0 |
|  | IC_025_=e ^ln(IC)-1.96(1/a+1/b+1/c+1/d)^0.5^ |  |
| MGPS | EBGM=a(a+b+c+d)/((a+c)/(a+b)) | EBGM05>2, N>0 |
|  | EBGM05=e ^ln(EBGM)-1.64(1/a+1/b+1/c+1/d)^0.5^ |  |

*a* number of reports of taking the target drug and having the target adverse reaction, *b* number of reports of taking the arget

drug with other adverse reactions*, c* number of reports of targeted adverse reactions while taking other drugs, *d* number of

reports of taking other drugs with other adverse reactions, *BCPNN* Bayesianconfidence Propagation Neural Network, *EBGM*

Empirical Bayesian Geometric Mean, *IC* Information Component, *PRR* Proportional Reporting Ratio, *ROR* Reporting

Dominance Ratio
